# Determination of 31 Polycyclic Aromatic Hydrocarbons in Plant Leaves Using Internal Standard Method with Ultrasonic Extraction–Gas Chromatography–Mass Spectrometry

**DOI:** 10.3390/toxics10110634

**Published:** 2022-10-22

**Authors:** Ming Yang, Shili Tian, Qingyang Liu, Zheng Yang, Yifan Yang, Peng Shao, Yanju Liu

**Affiliations:** 1 Institute of Analysis and Testing, Beijing Academy of Science and Technology (Beijing Center for Physical and Chemical Analysis), Beijing 100089, China; 2 College of Biology and the Environment, Nanjing Forestry University, Nanjing 210037, China; 3 Beijing Milu Ecological Research Center, Beijing 100076, China

**Keywords:** ultrasonic extraction, PAHs, internal standard method, gas chromatography–mass spectrometry

## Abstract

The method for the determination of 16 priority polycyclic aromatic hydrocarbons (PAHs) in plant leaves has been studied extensively, yet the quantitativemethod for measuring non-priority PAHs in plant leaves is limited. A method for the simultaneous determination of 31 polycyclic aromatic hydrocarbons (PAHs) in plant leaves was established using an ultrasonic extraction–gas chromatography–mass spectrometry–internal standard method. The samples of plant leaves were extracted with ultrasonic extraction and purified with solid-phase extraction columns. The PAHs were separated by using gas chromatography–mass spectrometry equipped with a DB-EUPAH capillary column (20 m × 0.18 mm × 0.14 μm) with a selective ion monitoring (SIM) detection mode, and quantified with an internal standard. The method had good linearity in the range of 0.005~1.0 μg/mL with correlation coefficients greater than 0.99, and the method detection limit and maximum quantitative detection limit were in the ranges of 0.2~0.7 μg/kg and 0.8~2.8 μg/kg, respectively. The method was verified with spiked recovery experiments. The average spiked recovery ranged from 71.0% to 97.6% and relative standard deviations (*n* = 6) were less than 14%. Herein, we established a quantitativemethod for the simultaneous determination of priority and non-priority PAHs in plant leaves using GC–MS. The method is highly sensitive and qualitatively accurate, and it is suitable for the determination of PAHs in plant leaves.

## 1. Introduction

Polycyclic aromatic hydrocarbons (PAHs) are produced by incomplete combustion or thermal decomposition of fossil fuels such as oil and coal and hydrocarbon-containing substances such as wood, natural gas, gasoline, heavy oil, organic polymer, paper, crop straw, tobacco, etc. [1,2,3]. Most of them are discharged into the ambient environment from emission sources including coal burning and vehicle emissions. PAHs further pollute water and soil with migration and transformation [4,5,6]. PAHs feature toxic characteristics including teratogenic, carcinogenic, and mutagenic traits, and have become a class of persistent organic pollutants that widely attracts concerns from the international academic community [1,7,8,9,10,11]. In China, especially in the northern region, PAH levels are extremely high because of the emissions from intensive industrial and domestic activities [12,13,14,15]. For instance, a study illustrated that the annual mean concentrations of atmospheric particulate BaP (a representative PAH) in cities of northern China were in the range of 1.1–14.43 ng/m^3^, which were greater than the recommended threshold value of 1.0 ng/m^3^ by the World Health Organization (WHO) [16]. The higher observed BaP in the atmospheric particulate matter could lead to a non-negligible health impact [16]. The estimation of health impacts associated with PAH inhalation exposure using incremental lifetime cancer risk (ILCR) was as high as 3.1 × 10^−5^ in 2007, which was much higher than the recommended safe level (10^−6^) and suggested urgent clean air interventions for PAHs [17].

Currently, the US Environmental Protection Agency (EPA) released the priority control of sixteen congeners of polycyclic aromatic hydrocarbons (PAHs) in the environment [8]. Among the sixteen congeners, benzo(a)pyrene (BaP) is categorized as a Group I carcinogen by the International Agency for Research on Cancer [6]. With the development of toxicological research on PAHs, the European Food Safety Agency (EFSA) re-proposed sixteen congeners of PAHs with greater toxicity in 2005 [18,19,20]. The list of sixteen congeners of PAHs from the EFSA includes eight congeners of PAHs in EPA priority control and eight new congeners [20]. In comparison, the other polycyclic aromatic compounds (PACs) receive less attention, including many compounds with stronger toxicity and unknown toxicity [6,19]. For example, non-priority PAHs have also been frequently detected, some of which exhibit stronger toxicity (10–100 times) than that of BaP, e.g., nitro polycyclic aromatic hydrocarbon [19]. The non-target analysis enables a comprehensive screening and identification of potential toxic PACs and provides support for accurate health risk assessments with comprehensive two-dimensional gas chromatography (GC × GC) and high-resolution mass spectrometry [1,7]. However, the non-target analysis of PACs has a disadvantage in the quantification.

The ecological remediation strategy has attracted attention as a cheap and sustainable environmental protection approach for reducing the high toxicity of polycyclic aromatic hydrocarbon pollution in environments [21,22,23,24]. Plants could be used as a filter for PAHs and the leaves of plants could be treated as a chemical reactor to remove the pollutants and reduce the concentrations of PAHs [25,26,27]. Several studies have found that the lipids inplant leaves could effectively enrich organic pollutants with high lipophilicity from the air in a variety of ways [28,29]. PAHs could stay in the leaves of plants for a relatively long time and be degraded under sunlight [24,29,30]. The studies on the levels, distribution, and transformation mechanism of priority PAHs in plant leaves have been extensive [22,24]. Yet, the quantitative method for determining non-priority congeners of PAHs is scarce. Some non-priority congeners of PAHs including coronene, retene, benzo(g,h,i)fluoranthene, and benzo(e)pyrene could be used as tracers for estimating source contributions to the ambient environment [3,5,31]. The qualitative methods for these non-priority congeners in the leaves of plants are helpful for understanding the associated source contributions from these non-priority congeners with stronger toxicity and unknown toxicity to protect public health. Thus, this study aims to establish a reliable quantitative method for determining 31 PAHs in plant leaves. The 31 congeners of PAHs include 16congeners on the priority pollutant list of the US EPA, 8congeners on the priority pollutant list of the EFSA, and 7congeners of non-priority PAHs used as source tracers.

## 2. Experimental

### 2.1. Reagents and Chemicals

A mixed standard of two substitutes (i.e., 2-fluorophenyl and terphenyl-d14) at 4000 mg/L was used for assessing the efficiencies of extraction processes. A mixed solution of five compounds (i.e., naphthalene-d8, acenaphthene-d10, phenanthrene-d10, chrysene-d12, perylene-d12) concentrated at 4000 mg/L wasadopted for internal standards. The mixed standards of 24 PAHs at 500 mg/L (AccuStandard, United States) including naphthalene, acenaphthylene, acenaphthene, fluorine, phenanthrene, anthracene, fluoranthene, pyrene, benzo(a)anthracene, chrysene, benzo(b)fluoranthene, benzo(k)fluoranthene, benzo(e)pyrene, indeno(1,2,3-cd)pyrene, dibenzo(a,h)anthracene, benzo(g,h,i)perylene, benzo(c)phenanthrene, 7,12-dimethylbenz(a)anthracene, benzo(j)fluoranthene, benzo(e)pyrene, 3-methylcholanthrene, picene, dibenzo(a,l)pyrene, and dibenzo(a,i)pyrene were usedfor a stock standard solution for PAH quantification in leaves. An amount of 50 mg/L of solution (AccuStandard, United States) containing coronene, perylene, cyclopenta(c,d)pyrene, and dibenzo(a,e)pyrene was used for a stock standard solution to quantify the levels of PAHs in leaves.

### 2.2. Sample Collections

Field samplings on leaf collections were conducted in West 3rd Ring North Road, Beijing (Figure S1). The sampling period was scheduled from February to March 2022. The leaves of 7 species of plants including *Berberis thunbergii*, Sabina Chinensis, Euonymus japonicas, Juniperus sabina, Buxus microphylla, Pinus tabuliformis, and *Pinus bungeana* were collected for PAH analysis. Four parallel leaves in one tree were collected and mixed as a respective sample. For each species, four respective samples were collected. A total of 28 respective samples were collected for PAH analysis.

### 2.3. Extraction Procedure

About 200 g of leaf samples was collected and fully crushed to a powder with a high-speed grinder. Then, 100 g of leaf powder was put into a clean sample bag sealed with a label and kept at 18 °C before analysis. An amount of 5 g of the prepared leaf powder was placed into a 100 mL centrifuge tube and mixed with 10 μL of internal standard solution (20 μg/mL). Then, 5 g anhydrous sodium sulfate and 50 mL solution of n-hexane and dichloromethane (1:1, *v*/*v*) were added to the 100 mL centrifuge tube and mixed using a vortex mixer for 1 min. Three methods including Soxhlet extraction, ultrasonic extraction, and accelerated solvent extraction were carried out to select the optimized extraction method. For Soxhlet extraction, the solution was extracted using the Soxhlet extraction apparatus for 3 h at 80 °C. For ultrasonic extraction, the solution was ultrasonically extracted for 30 min in a water bath with a temperature lower than 30 °C. Accelerated solvent extraction was performed using Dionex ASE 350 at the temperature of 100 °C and under the pressure of 103.4 Mpa. After the extraction, the solution was centrifuged at 10,000 r/min for 3 min. The extraction processes were carried out twice in parallel. The supernatants were taken out and kept in a vessel for further experiments. A total of 5 g of anhydrous sodium sulfate was added to a layer of glass wool placed on the glass funnel to remove the water in the supernatants. The supernatants were filtered through 5 g of anhydrous sodium sulfate and dropped into the concentration vessel. Next, about 2 mL of the mixed solution of n-hexane and dichloromethane (1:1, *v*/*v*) was added to the layer of glass wool and placed on the glass funnel for washing. The washing processes were performed 3 times. Finally, after the removal of water, the extracted solutions were collected in the concentration vessel for further treatment.

At room temperature, the extracted solutions were concentrated to 1–2 mL using a multi-sample parallel evaporator (Q-101, BUCHI, Flawil, Switzerland). Then, 10 mL of n-hexane was added into the concentration vessel and concentrated continually. During the processes, the solvent for the exacted solution was changed to n-hexane. A total of 2 mL of the extracted solution was kept for further purification using solid-phase extraction. Solid-phase extraction was carried out using a solid extraction column of 800 mg silica gel and 1200 mg neutral alumina. The extracted solutions were pumped through the solid extraction column. Then, 2 mL of dichloromethane and 8 mL of n-hexane were passed through the solid extraction column as the washing solution. The washing processes were repeated 3 times. After the purification by solid-phase extraction, the solution was concentrated to 0.5–1 mL using the evaporator. A total of 20 μg/mL of internal standard was added into the concentrated extraction solution for further quantitative analysis.

### 2.4. Instrumentations

A gas chromatography–mass spectrometry QP2010Ultra (GC–MS QP2010Ultra, Shimadzu, Kyoto, Japan) was used for PAH quantification. The GC–MS QP2010Ultra was equipped with a DB-EUPAH capillary column (20 m × 0.18 mm × 0.14 μm, Agilent, Palo Alto, California, USA). The temperature was programmed at the initial temperature of 70 °C held for 2 min, and then increased to 280 °C with a heating rate of 10 °C/min, and kept at 280 °C for 5 min. Next, the temperature was increased to 320 °C at a heating rate of 5 °C/min and kept constant for 10 min. The temperature of the injection port was set to 260 °C. The carrier gas was maintained at a flow rate of 1.0 mL/min in high-purity helium. The injection volume was 1.0 μL without split injection. The detection of mass spectrometry was performed at an electron-impact (EI) ion source with electron energy of 70 eV in selected ion monitoring (SIM) mode. The temperatures of the ion source, fourth stage rod, and transmission line were set to be 240 °C, 150 °C, and 280 °C, respectively.

### 2.5. Statistical Analysis

Based on the results from at least three independent analytical experiments conducted with GC–MS, summary statistics for data are shown as the mean and standard deviation (SD) for each sample in the study at 95% confidence. Student’s t-tests were applied for calculating the mean differences in extraction efficiencies of thesolid-phase column across groups. *p*-values lower than 0.05 indicated significant differences. All statistical analyses were carried out with SPSS V20.0.

## 3. Results and Discussion

### 3.1. Optimization of Detection Conditions

#### 3.1.1. Selection of Internal Standards and Substitutes

**Figure 1:**
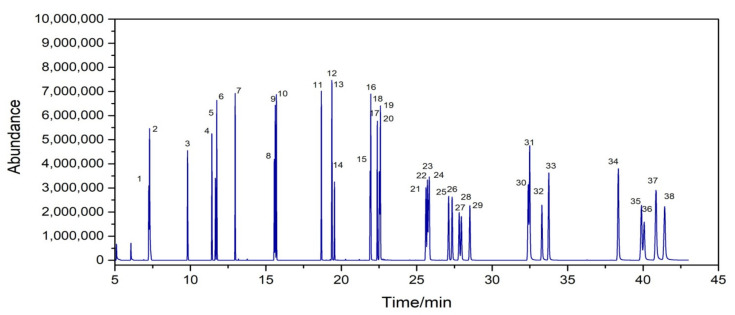
Select ion monitoring chromatogram of 31 PAHs,2 substitutes, and 5 internal standards. 1. Naphthalene-d8, 2. Naphthalene, 3. 2-Fluorobiphenyl, 4. Acenaphthylene, 5. Acenaphthylene-d10, 6. Acenaphthene, 7. Fluorene, 8. Phenanthrene-d10, 9. Phenanthrene, 10. Anthracene, 11. Fluoranthene, 12. Retene, 13. Pyrene, 14. Terphenyl-d14, 15. Benzo(g,h,i)perylene, 16. Benzo(c)phenanthrene, 17. Benz(a)anthracene, 18. Chrysene-d12, 19. Cyclopenta(c,d)pyrene, 20. Chrysene, 21. Benzo(b)fluoranthene, 22. Benzo(k)fluoranthene, 23. 7,12-Dimethylbenz(a)anthracene, 24. Benzo(j)fluoranthene, 25. Benz(e)pyrene, 26. Benz(a)pyrene, 27. Perylene-d12, 28. Perylene, 29. 3-Methylcholanthrene, 30. Indeno(1,2,3-cd)pyrene, 31. Dibenz(a,h)anthracene, 32. Picene, 33. Benzo(g,h,i)perylene, 34. Dibenz(a,l)pyrene, 35. Dibenz(a,e)pyrene, 36. Coronene, 37. Dibenz(a,i)pyrene, 38. Dibenz(a,h)pyrene.

Since the interferences of the environmental matrix on the detections of PAHs in samples and the concentrations of PAHs are at trace levels [22], the determination of PAHs in environmental samples with the use of GC–MS should be carried out after the pretreatment of environmental samples to remove matrix effects. PAHs have a class of semi-volatile organic compounds including naphthalene, acenaphthylene, and acenaphthene, which feature the characteristics of small molecular weight, low boiling point, and are easy to sublimate during pretreatment procedures [11]. The loss proportions (i.e., recovery rate) of semi-volatile organic compounds during the pretreatment procedure should be estimated to ensure the reliability and accuracy of PAHs in environmental samples [32]. The use of substitutes could aid in tracking the recovery rate during sample pretreatment before GC–MS [33]. In this study, known concentrations of 2-fluorophenyl and triphenyl-d14 (20 μg/mL) were selected as substitutes for the determination of the recovery rate of PAHs in environment samples during the pretreatment procedure because they have similar structure and nature. In addition, the internal standards could eliminate the interferences of the environmental matrix on quantitative analysis of GC–MS. Thus, known levels (20 μg/mL) of naphthalene-d8, acenaphthene-d10, phenanthrene-d10, chrysene-d12, and perylene-d12 were used in this study as internal standards for the determination of PAHs because these internal standards are similar in structure and properties to analyte, and have chromatographically similar retention times that can be fully separated on the chromatographic column (Figure 1). The internal standards do not react with each other and do not exist in the actual sample, which meets the basic requirements criteria for substitutes and internal standards in sample analysis [33].

#### 3.1.2. Selection of Quantitative Ion

According to the mass spectrum characteristics of the target compound, the characteristic ions with high abundance and of the high-mass end are selected for selective ion scanning mode (SIM) determination to reduce interference and improve the selectivity and sensitivity of the method [34]. In this study, the base peak ion of each target component was selected as the quantitative ion, and the two characteristic ions at the high-mass end were selected as the mass spectrum conditions of the auxiliary qualitative ions (Table 1). Based on the peak area of the quantitative ions, the content of PAHs in plant leaves could be accurately analyzed by using the internal standard method.

#### 3.1.3. Calibrations and Sensitivity of Instruments

The calibrations of GC–MS were assessed by in-house validation in terms of the regression coefficient, linear range, analyte detectability, and relative standard deviation using a mixed standard solution of PAHs. Linearity was assessed by adding appropriate volumes of standard solutions at concentrations ranging from 0.005–1.0 μg/mL. The correlation coefficients for all analytes were higher than 0.99 (Table 2). The sensitivity of the instruments was assessed in terms of limits of detection (LODs), which were calculated as three times the standard deviation of background noise divided by the slope of each calibration graph. LODs for all analytes were in the range of 0.1 to 0.5 ng/mL. The precision of the method, as relative standard deviation (RSD), was determined by analyzing 11 samples of mixed standard solution spiked with PAHs at three different concentrations. Analyses were performed on the same day (within-day precision) or seven different days (between-day precision). The within-day precision and between-day precision ranged from 2.1–6.3% and 3.5–7.2%., respectively.

### 3.2. Optimization of Solid-Phase Extraction Conditions

#### 3.2.1. Selection of Extraction Method

**Figure 2:**
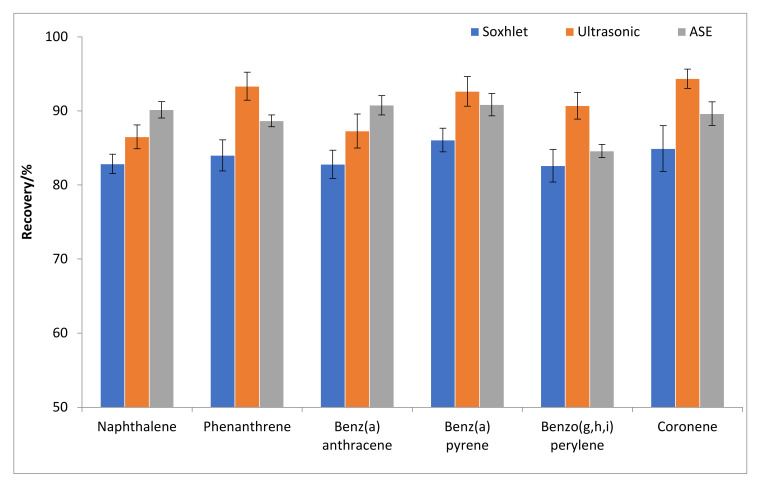
The extraction recoveries of selected PAHs using three different methods including Soxhlet extraction, ultrasonic extraction, and accelerated solvent extraction (ASE).

Three extraction methods including ultrasonic extraction, Soxhlet extraction, and accelerated solvent extraction were adopted for the determination of polycyclic aromatic hydrocarbons in plant leaves [35,36]. The extraction efficiencies across the three methods (i.e., ultrasonic extraction, Soxhlet extraction, and accelerated solvent extraction) were compared through sample spiked recovery tests. As shown in Figure 2, no significant extraction efficiencies for the medium-molecular-weight and high-molecular-weight PAHs were observed across these three methods. In contrast, higher extraction efficiencies for low-molecular-weight PAHs (e.g., naphthalene) were found using accelerated solvent extraction compared to those using the other two methods because the good sealing performance of the fast solvent extraction tank leads to the minor loss of naphthalene during the extraction process. Considering that ultrasonic extraction is more suitable for the simultaneous processing of large batches of leaf samples than Soxhlet extraction and accelerated solvent extraction. Thus, ultrasonic extraction was selected for the extraction of samples in this study.

#### 3.2.2. Selection of Solid-Phase Extraction Column

To choose the solid-phase extraction column for minimizing the environmental matrix on the detection of GC–MS, four kinds of solid-phase extraction columns including a carbon/NH_2_solid-phase extraction column (2 g/6 mL), Florisilsolid-phase extraction column (2 g/6 mL), alumina-N solid-phase extraction column (2 g/6 mL), and composite solid-phase extraction column (800 mg silica gel and 1200 mg neutral alumina, 2 g/6 mL) were adopted. After loading 1 mL of 31 target PAH compounds at 1.0 μg/mL on the columns, the recoveries of the 31 target PAH compounds were found to be higher than 90% with the use of the Florisilsolid-phase extraction column, alumina-N solid-phase extraction column, and composite solid-phase extraction column, while the recoveries of the 31 target PAH compounds were lower than 20% with the use of the carbon/NH_2_solid-phase extraction column. The surfaces of the carbon/NH_2_solid-phase extraction column feature a positive six-membered ring structure, which exhibits a strong affinity for planar molecules such as PAHs [37]. The elution solution of n-hexane and dichloromethane was incapable of washing target PAH compounds from the carbon/NH_2_solid-phase extraction column.

**Figure 3:**
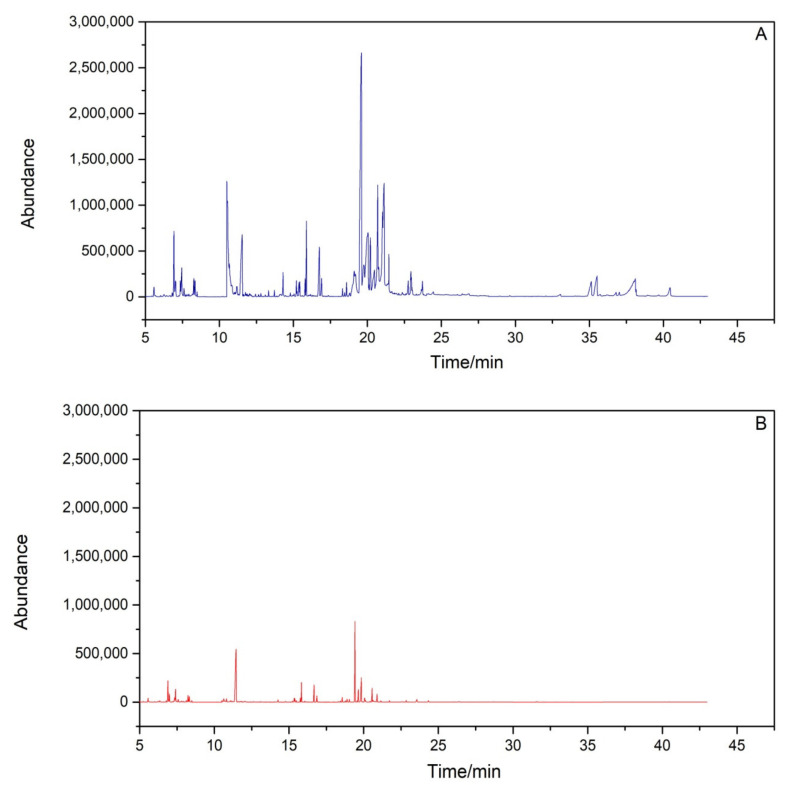
Purificationeffect of environmental matrixes using solid-phase extraction on total ion chromatogram of GC–MS. (**A**) unpurified sample, (**B**) purified sample with solid-phase extraction.

Because large amounts of pigments, lipids, waxes, and other impurities exist in plant leaf samples that interface the detections of target PAH compounds using GC–MS [21,36], the purification effects of three solid-phase extraction columns including the Florisilsolid-phase extraction column, alumina-N solid-phase extraction column, and composite solid-phase extraction column were also investigated. As shown in Figure 3, the composite solid-phase extraction column showed better purification efficiencies for the impurities (e.g., pigments, lipids, and wax in leaves) than the other two solid-phase extraction columns (i.e., Florisilsolid-phase extraction column and alumina-N solid-phase extraction column). Therefore, the composite solid-phase extraction column (800 mg silica gel and 1200 mg neutral alumina, 2 g/6 mL) was selected to purify the sample in this study.

#### 3.2.3. Selection of Elution Volume

**Figure 4:**
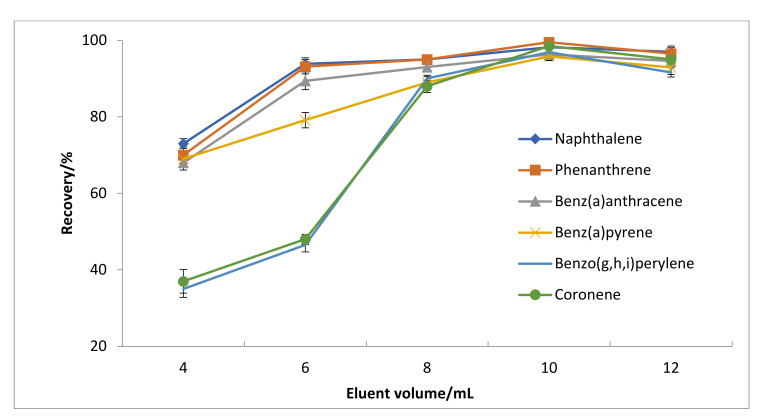
Effects of elution volume on recoveries of PAHs using solid-phase extraction.

In this study, the recoveries of 31 polycyclic aromatic hydrocarbons were investigated with the use of different total elution volumes (e.g., 4, 6, 8, 10, and 12 mL) of dichloromethane and hexane (1:4, *v*/*v*) solution. The results indicated that the recoveries of individual PAHs tend to be stable at 90% when 10 mL or 12 mL of total elution volumes was added (Figure 4). Thus, a total elution volume of 10 mL of dichloromethane and hexane (1:4, *v*/*v*) solution was chosen as the optimized elution volume in solid-phase extraction.

### 3.3. Method Validation

Under the optimal extraction and determination conditions, the linear range, detection limit, and lower limit of the method were verified. As shown in Table 2, the linearity was in the range of 0.005–1.0 μg/mL, and the correlation coefficient is greater than 0.99. The detection limits and the quantification limits of 31 target PAH compounds fell in the ranges of 0.2–0.7 ng/g and 0.8–2.8 ng/g. The standard addition recoveries were performed to test the precision of the established method. Under the six parallel determinations, the average recoveries of 31 target compounds were observed to be in the range of 71.0–97.6% with the relative standard deviation in the range of 0.5–13.5%. The quality of the established method meets the acceptance criteria of internal quality control procedures for PAHs by SW-846 Method 8310 (65–125% of expected value for PAHs and surrogates) [8].

The recovery results show that this method is accurate and reliable, and is suitable for the analysis of 31 PAHS in leaves (Table 3).

### 3.4. Detection of PAHs in Real Leaf Samples

By using the established method, the levels of 31 PAHs in the leaves of 7 plants including *Berberis thunbergii*, *Sabina Chinensis*, *Euonymus japonicas*, *Juniperus sabina*, *Buxus microphylla*, *Pinus tabuliformis*, and *Pinus bungeana* were analyzed. The results indicated that the total levels of 31 PAHs in the leaves of 7 plants were found to be in the range of 71.6 to 230 ng/g. The highest level of total PAHs was observed in *Juniperus Sabina* (230 ± 21.4 ng/g), followed by *Sabina Chinensis* (194 ± 17.8 ng/g), Pinus bungeana (114 ± 10.8 ng/g), Buxus microphylla (102 ± 13.4 ng/g), *Pinus tabuliformis* (93.0 ± 5.9 ng/g), and *Berberis thunbergii* (90.8 ± 1.2 ng/g) (Table 4). In addition, some congeners of PAHs, without listing the priority control list of the EPA including retene, benzo(g,h,i)perylene, and cyclopenta(c,d)pyrene, were detected in the samples with a concentration in the range of 2.3–6.6 ng/g.Retene is regarded as the incomplete combustion product of conifers, while cyclopentene(c,d)pyrene is used as a marker of gasoline vehicle emission. The occurrences of retene and cyclopenta(c,d)pyrene detected in the leaves of seven plants located at West 3rd Ring North Road, Beijing indicated that the emission sources including incomplete combustion of conifers and gasoline vehicle emissions contributed to air pollution in urban Beijing during cold periods. The total concentration of low-molecular-weight PAHs (2–3 rings) accounted for the highest fraction of the total PAHs in the range of 44–78%, followed by medium-molecular-weight PAHs ( 4rings) (20–40%), and high-molecular-weight PAHs (5–6 rings) (12–20%). This is because low-molecular-weight PAHs mainly exist in the form of the gas phase and high-molecular-weight PAHs mainly exist in the form of the particulate phase, while medium-molecular-weight PAHs coexist in both the gas phase and particulate phase [14]. PAHs in the atmosphere are found to enter the stomata on the surfaces of leaves through gas exchange [36]. Therefore, low-molecular-weight PAHs could be more easily absorbed by leaves than medium-molecular-weight and high-molecular-weight PAHs, which was consistent with prior results [38,39,40]. Mukhopadhyay et al. [36] reviewed ~50 recent studies on the determination of 16 priority PAHs in the leaves of plants across the world. The concentrations of a total of 16 priority PAHs were observed to be in the range of 4–2610 ng g^−1^. The levels of a total of 16 priority PAHs in our study were comparable with those observed in other sites. In addition, our study presents the levels of non-priority PAHs in the leaves of seven plants using the established method. It is important to note that the variations in the levels of PAHs across different sites are supposed to depend on many factors including local meteorological parameters, air compartment structure, type of plants, and half-lives of individual PAHs [29,30,38,39,40]. Further studies are certainly needed to fully understand how the different factors affect the levels of priority and non-priority PAHs in different plants, resulting in diverse toxicological effects.

## 4. Conclusions

In this study, we improved the qualitative method to enable the determination of priority and non-priority PAHs in plant leaves using an internal standard method coupled with ultrasonic extraction and GC–MS simultaneously. The established method is found to be highly sensitive with a detection limit and a quantitative detection limit ranging from 0.2~0.7 μg/kg and 0.8~2.8 μg/kg, respectively. The accuracy of this qualitative method for the determination of PAHs in leaves was verified with spiked recovery experiments with average spiked recovery in the range of 71.0% to 97.6% and relative standard deviations (n = 6) less than 14%. This method could be applied in high-throughput and rapid quantitative detection of priority and non-priority PAHs in plant leaves. This method provides a robust method for measuring the levels of priorityand non-priority PAHs in plant leaves qualitatively and, thereby, understanding the distribution characteristics, migration, and transformation mechanism of non-priority PAHs between the interfaces of plant leaves and air.

## Figures and Tables

**Table 1:** Retention times, quantitative ions, and qualitative ions of PAHs.

No.	Compound	Formula	Number of Benzene Ring	Retention Time (min)	Quantitative Ion (m/z)	Qualitative Ions (m/z)	
1	Naphthalene-d8	C_10_D_8_	-	7.195	136	108	154
2	Naphthalene	C_10_H_8_	2	7.251	128	127	129
3	2-Fluorobiphenyl	C_12_H_9_F	-	9.815	172	171	170
4	Acenaphthylene	C_12_H_8_	3	11.405	152	151	153
5	Acenaphthylene-d10	C_12_D_10_	-	11.640	162	160	163
6	Acenaphthene	C_12_H_10_	3	11.735	154	153	152
7	Fluorene	C_13_H_10_	3	12.955	166	165	167
8	Phenanthrene-d10	C_14_D_10_	-	15.545	188	189	160
9	Phenanthrene	C_14_H_10_	3	15.610	178	179	176
10	Anthracene	C_14_H_10_	3	15.685	178	179	176
11	Fluoranthene	C_16_H_10_	4	18.665	202	200	101
12	Retene	C_18_H_18_	3	19.35	219	204	234
13	Pyrene	C_16_H_10_	4	19.368	202	200	101
14	Terphenyl-d14	C_18_D_14_	-	19.550	244	245	243
15	Benzo(g,h,i)perylene	C_18_H_10_	4	21.895	226	224	113
16	Benzo(c)phenanthrene	C_18_H_12_	4	21.940	228	226	227
17	Benz(a)anthracene	C_18_H_12_	4	22.370	228	226	229
18	Chrysene-d12	C_18_D_12_	-	22.505	240	236	238
19	Cyclopenta(c,d)pyrene	C_18_H_10_	4	22.531	226	224	227
20	Chrysene	C_18_H_12_	4	22.575	228	226	229
21	Benzo(b)fluoranthene	C_20_H_12_	5	25.585	252	253	250
22	Benzo(k)fluoranthene	C_20_H_12_	5	25.680	252	253	250
23	7,12-Dimethylbenz(a)anthracene	C_20_H_16_	4	25.750	256	241	239
24	Benzo(j)fluoranthene	C_20_H_12_	5	25.800	252	253	250
25	Benz(e)pyrene	C_20_H_12_	5	27.080	252	253	250
26	Benz(a)pyrene	C_20_H_12_	5	27.315	252	253	250
27	Perylene-d12	C_20_D_12_	-	27.785	264	260	265
28	Perylene	C_20_H_12_	5	27.925	252	253	250
29	3-Methylcholanthrene	C_21_H_16_	5	28.490	268	253	252
30	Indeno(1,2,3-cd)pyrene	C_22_H_12_	6	32.355	276	275	274
31	Dibenz(a,h)anthracene	C_22_H_14_	5	32.460	278	276	279
32	Picene	C_22_H_14_	5	33.265	278	276	279
33	Benzo(g,h,i)perylene	C_22_H_12_	6	33.720	276	275	274
34	Dibenz(a,l)pyrene	C_24_H_14_	6	38.325	300	303	302
35	Dibenz(a,e)pyrene	C_24_H_14_	6	39.840	302	150	300
36	Coronene	C_24_H_12_	7	40.015	300	301	150
37	Dibenz(a,i)pyrene	C_24_H_14_	6	40.800	300	303	302
38	Dibenz(a,h)pyrene	C_24_H_14_	6	41.365	300	303	302

**Table 2:** Linear ranges, linear equations, correlation coefficients, limits of detection limit, and limits of quantification of PAHs.

Compound	Linear Range (μg/mL)	Linear Equation	Correlation Coefficient (*R^2^*)	Limit of Detection (ng/g)	Limit of Quantification (ng/g)
Naphthalene	0.005–1.0	*y* = 8.46*x* − 0.04	0.99	0.3	1.2
Acenaphthylene	0.005–1.0	*y* = 5.78*x* − 0.03	0.99	0.4	1.6
Acenaphthene	0.005–1.0	*y* = 7.92*x* − 0.05	0.99	0.4	1.6
Fluorene	0.005–1.0	*y* = 9.52*x* − 0.06	0.99	0.7	2.8
Phenanthrene	0.005–1.0	*y* = 6.84*x* − 0.04	0.99	0.3	1.2
Anthracene	0.005–1.0	*y* = 7.10*x* − 0.05	0.99	0.6	2.4
Fluoranthene	0.005–1.0	*y* = 8.03*x* − 0.06	0.99	0.3	1.2
Retene	0.005–1.0	*y* = 2.16*x* − 0.02	0.99	0.5	2.0
Pyrene	0.005–1.0	*y* = 8.71*x* − 0.07	0.99	0.3	1.2
Benzo(g,h,i)perylene	0.005–1.0	*y* = 7.96*x* − 0.03	0.99	0.2	0.8
Benzo(c)phenanthrene	0.005–1.0	*y* = 6.99*x* + 0.04	0.99	0.3	1.2
Benz(a)anthracene	0.005–1.0	*y* = 9.48*x* − 0.07	0.99	0.4	1.6
Cyclopenta(c,d)pyrene	0.005–1.0	*y* = 6.27*x* − 0.06	0.99	0.3	1.2
Chrysene	0.005–1.0	*y* = 9.62*x* − 0.05	0.99	0.5	2.0
Benzo(b)fluoranthene	0.005–1.0	*y* = 8.54*x* − 0.07	0.99	0.4	1.6
Benzo(k)fluoranthene	0.005–1.0	*y* = 9.20*x* − 0.07	0.99	0.4	1.6
7,12-Dimethylbenz(a)anthracene	0.005–1.0	*y* = 3.65*x* − 0.03	0.99	0.2	0.8
Benzo(j)fluoranthene	0.005–1.0	*y* = 8.60*x* − 0.04	0.99	0.2	0.8
Benz(e)pyrene	0.005–1.0	*y* = 7.44*x* − 0.04	0.99	0.3	1.2
Benz(a)pyrene	0.005–1.0	*y* = 7.23*x* − 0.06	0.99	0.4	1.6
Perylene	0.005–1.0	*y* = 6.71*x* − 0.05	0.99	0.2	0.8
3-Methylcholanthrene	0.005–1.0	*y* = 4.48*x* − 0.05	0.99	0.3	1.2
Indeno(1,2,3-cd)pyrene	0.005–1.0	*y* = 8.78*x* − 0.10	0.99	0.3	1.2
Dibenz(a,h)anthracene	0.005–1.0	*y* = 9.76*x* − 0.10	0.99	0.5	2.0
Picene	0.005–1.0	*y* = 5.90*x* − 0.05	0.99	0.2	0.8
Benzo(g,h,i)perylene	0.005–1.0	*y* = 9.71*x* − 0.07	0.99	0.2	0.8
Dibenz(a,l)pyrene	0.005–1.0	*y* = 7.84*x* − 0.07	0.99	0.5	2.0
Dibenz(a,e)pyrene	0.005–1.0	*y* = 8.84*x* − 0.07	0.99	0.7	2.8
Coronene	0.005–1.0	*y* = 8.17*x* − 0.03	0.99	0.6	2.4
Dibenz(a,i)pyrene	0.005–1.0	*y* = 8.51*x* − 0.14	0.99	0.3	1.2
Dibenz(a,h)pyrene	0.005–1.0	*y* = 6.30*x* − 0.12	0.99	0.6	2.4

**Table 3:** Spiked recoveries and precision of PAHs in leaves (*n* = 6).

Compound	Spiked (ng/g)	Recovery/%	RSD/%	Compound	Spiked (ng/g)	Recovery/%	RSD/%
Naphthalene	2	85.4	5.8	7,12-Dimethylbenz(a) anthracene	2	71.8	4.5
	20	87.8	9.8		20	76.8	8.6
	120	97.6	4.2		120	76.8	3.0
Acenaphthylene	2	74.2	8.2	Benzo(j)fluoranthene	2	72.3	4.6
	20	79.2	11.1		20	75.3	8.1
	120	72.4	4.6		120	76.2	3.9
Acenaphthene	2	89.2	6.5	Benz(e)pyrene	2	74.2	8.2
	20	90.4	10.5		20	73.2	10.6
	120	83.0	7.8		120	81.6	2.3
Fluorene	2	88.2	12.1	Benz(a)pyrene	2	74.9	8.7
	20	78.4	4.5		20	75.8	10.4
	120	88.0	7.8		120	83.9	2.1
Phenanthrene	2	85.2	6.4	Perylene	2	72.7	4.4
	20	84.9	10.2		20	73.8	10.8
	120	86.7	0.5		120	82.7	2.5
Anthracene	2	79.7	11.1	3-Methylcholanthrene	2	85.5	5.7
	20	77.1	10.7		20	86.8	7.8
	120	86.1	1.1		120	95.5	1.9
Fluoranthen	2	85.4	6.2	Indeno(1,2,3-cd)pyrene	2	92.9	5.9
	20	88.0	10.5		20	96.0	6.8
	120	86.9	2.6		120	78.4	1.9
Retene	2	79.2	10.5	Dibenz(a,h)anthracene	2	92.3	9.3
	20	81.7	11.2		20	96.5	7.0
	120	77.4	11.9		120	73.5	3.6
Pyren	2	85.4	6.3	Picene	2	74.4	4.6
	20	85.0	11.3		20	78.5	9.7
	120	83.6	3.9		120	86.0	7.0
Benzo(g,h,i)perylene	2	72.3	6.1	Benzo(g,h,i)perylene	2	73.4	5.8
	20	76.7	11.2		20	71.8	2.5
	120	88.4	1.1		120	75.9	3.5
Benzo(c)phenanthrene	2	72.1	8.5	Dibenz(a,l)pyrene	2	71.0	13.4
	20	80.2	10.7		20	72.5	10.4
	120	89.9	1.7		120	92.3	5.6
Benz(a)anthracene	2	72.5	9.1	Dibenz(a,e)pyrene	2	80.6	13.5
	20	76.4	10.9		20	87.3	5.1
	120	84.1	2.3		120	93.0	5.1
Cyclopenta(c,d)pyren	2	78.3	6.2	Coronene	2	81.9	11.0
	20	89.8	10.1		20	83.2	6.2
	120	89.1	10.0		120	91.0	6.3
Chrysene	2	70.3	11.8	Dibenz(a,i)pyrene	2	83.5	5.2
	20	75.7	11.3		20	85.3	3.7
	120	81.0	4.2		120	89.3	6.5
Benzo(b)fluoranthene	2	80.2	8.7	Dibenz(a,h)pyrene	2	76.9	12.5
	20	80.5	8.3		20	89.0	4.2
	120	76.0	3.9		120	94.4	5.0
Benzo(k)fluoranthene	2	72.0	8.9				
	20	80.2	8.4				
	120	75.1	4.0				

**Table 4:** Concentration (average ± SD) of PAHs in different plant leaves (ng/g).

Compound	*Berberis thunbergii*	*Sabina chinensis*	*Euonymus* *japonicus*	*Juniperus sabina*	*Buxus microphylla*	*Pinus* *tabuliformis*	*Pinus bungeana*
Naphthalene	7.3 ± 0.9	27.3 ± 3.9	4.1 ± 1.0	107 ± 18.8	5.8 ± 0.2	18.1 ± 1.2	30.4 ± 3.9
Acenaphthylene	2.2 ± 0.2	2.7 ± 0.8	N.D.	6.0 ± 0.7	2.5 ± 0.1	2.3 ± 0.3	2.3 ± 0.1
Acenaphthene	N.D.	2.5 ± 0.3	N.D.	3.3 ± 0.9	N.D.	N.D.	N.D.
Fluorene	6.1 ± 0.9	10.5 ± 2.2	3.7 ± 0.7	13.4 ± 0.8	5.7 ± 0.5	8.7 ± 0.8	13.9 ± 2.3
Phenanthrene	26.7 ± 3.1	27.1 ± 6.2	19.4 ± 2.5	41.3 ± 8.2	27.9 ± 2.7	31.4 ± 3.5	37.6 ± 5.3
Anthracene	2.0 ± 0.0	14.0 ± 2.2	2.2 ± 0.3	2.4 ± 0.2	2.3 ± 0.4	2.2 ± 0.1	2.1 ± 0.0
Fluoranthene	9.7 ± 0.8	20.4 ± 4.1	7.2 ± 2.1	18.3 ± 1.7	15.7 ± 1.2	10.4 ± 0.9	10.3 ± 2.3
Retene	2.8 ± 0.2	4.0 ± 0.3	2.8 ± 0.1	3.4 ± 0.3	3.3 ± 0.1	2.8 ± 0.2	3.2 ± 0.2
Pyrene	6.0 ± 0.5	10.8 ± 1.6	6.8 ± 1.0	9.5 ± 0.5	10.8 ± 1.7	6.8 ± 0.6	9.1 ± 1.4
Benzo(g,h,i)perylene	N.D.	3.8 ± 0.4	2.3 ± 0.4	N.D.	2.5 ± 0.3	N.D.	N.D.
Benzo(c)phenanthrene	N.D.	N.D.	N.D.	N.D.	N.D.	N.D.	N.D.
Benz(a)anthracene	2.0 ± 0.0	3.3 ± 0.5	2.4 ± 0.2	2.2 ± 0.1	2.5 ± 0.3	N.D.	N.D.
Cyclopenta(c,d)pyrene	4.1 ± 0.2	6.6 ± 1.7	4.4 ± 0.6	4.9 ± 0.4	3.8 ± 0.6	3.3 ± 0.1	2.6 ± 0.1
Chrysene	4.7 ± 0.4	13.2 ± 3.1	5.2 ± 1.1	9.2 ± 1.6	5.2 ± 1.2	3.5 ± 0.1	N.D.
Benzo(b)fluoranthene	2.9 ± 0.5	7.0 ± 1.1	N.D.	N.D.	3.1 ± 0.7	N.D.	N.D.
Benzo(k)fluoranthene	2.2 ± 0.0	2.8 ± 0.5	N.D.	N.D.	2.2 ± 0.1	N.D.	N.D.
7,12-Dimethylbenz(a)anthracene	N.D.	N.D.	N.D.	N.D.	N.D.	N.D.	N.D.
Benzo(j)fluoranthene	N.D.	2.4 ± 0.2	N.D.	N.D.	N.D.	N.D.	N.D.
Benz(e)pyrene	2.4 ± 0.2	11.2 ± 2.4	2.8 ± 0.7	2.3 ± 0.3	3.2 ± 0.8	N.D.	N.D.
Benz(a)pyrene	2.3 ± 0.1	2.7 ± 0.3	2.5 ± 0.3	2.4 ± 0.3	2.4 ± 0.2	N.D.	N.D.
Perylene	N.D.	7.9 ± 0.8	N.D.	N.D.	N.D.	N.D.	N.D.
3-Methylcholanthrene	N.D.	N.D.	N.D.	N.D.	N.D.	N.D.	N.D.
Indeno(1,2,3-cd)pyrene	2.9 ± 0.5	2.9 ± 0.7	2.5 ± 0.4	2.7 ± 0.5	2.8 ± 0.5	N.D.	N.D.
Dibenz(a,h)anthracene	2.1 ± 0.0	N.D.	N.D.	N.D.	N.D.	3.5 ± 0.2	2.1 ± 0.0
Picene	N.D.	7.4 ± 1.3	N.D.	N.D.	N.D.	N.D.	N.D.
Benzo(g,h,i)perylene	2.4 ± 0.3	3.4 ± 0.8	3.3 ± 0.6	2.3 ± 0.3	N.D.	N.D.	N.D.
Dibenz(a,l)pyrene	N.D.	N.D.	N.D.	N.D.	N.D.	N.D.	N.D.
Dibenz(a,e)pyrene	N.D.	N.D.	N.D.	N.D.	N.D.	N.D.	N.D.
Coronene	N.D.	N.D.	N.D.	N.D.	N.D.	N.D.	N.D.
Dibenz(a,i)pyrene	N.D.	N.D.	N.D.	N.D.	N.D.	N.D.	N.D.
Dibenz(a,h)pyrene	N.D.	N.D.	N.D.	N.D.	N.D.	N.D.	N.D.
LMW(2~3 Rings)	47.1 ± 3.3	88.1 ± 10.3	32.1 ± 6.3	176.5 ± 20.4	47.5 ± 6.6	65.5 ± 5.4	89.5 ± 8.4
MMW(4 Rings)	26.5 ± 0.8	58.3 ± 9.6	28.4 ± 4.5	43.8 ± 4.8	40.8 ± 5.2	24.0 ± 3.1	22.4 ± 2.6
HMW(5~6 Rings)	17.3 ± 2.1	47.6 ± 8.9	11.1 ± 2.3	9.7 ± 2.1	13.7 ± 1.1	3.5 ± 1.2	2.1 ± 0.9
ΣPAHs	90.8 ± 1.2	194 ± 17.8	71.6 ± 9.9	230 ± 21.4	102 ± 13.4	93.0 ± 5.9	114 ± 10.8

N.D.: Lower than the detection limits (<0.2 ng/g); LMW: Low molecular weight; MMW: Medium molecular weight; HMW: High molecular weight.

## Data Availability

The data materials are shown in the main text and can also be acquired upon request from the corresponding author.

## Supplementary Materials

The following supporting information can be downloaded at: https://www.mdpi.com/article/10.3390/toxics10110634/s1, Figure S1: The location of tree leaves collected.
