# Supplementary figures and images for: Determination of 31 Polycyclic Aromatic Hydrocarbons in Plant Leaves Using Internal Standard Method with Ultrasonic Extraction–Gas Chromatography–Mass Spectrometry
Source: Toxics. 2022 Oct 22;10(11):634. doi: 10.3390/toxics10110634 (PMC9698594; doi:10.3390/toxics10110634)

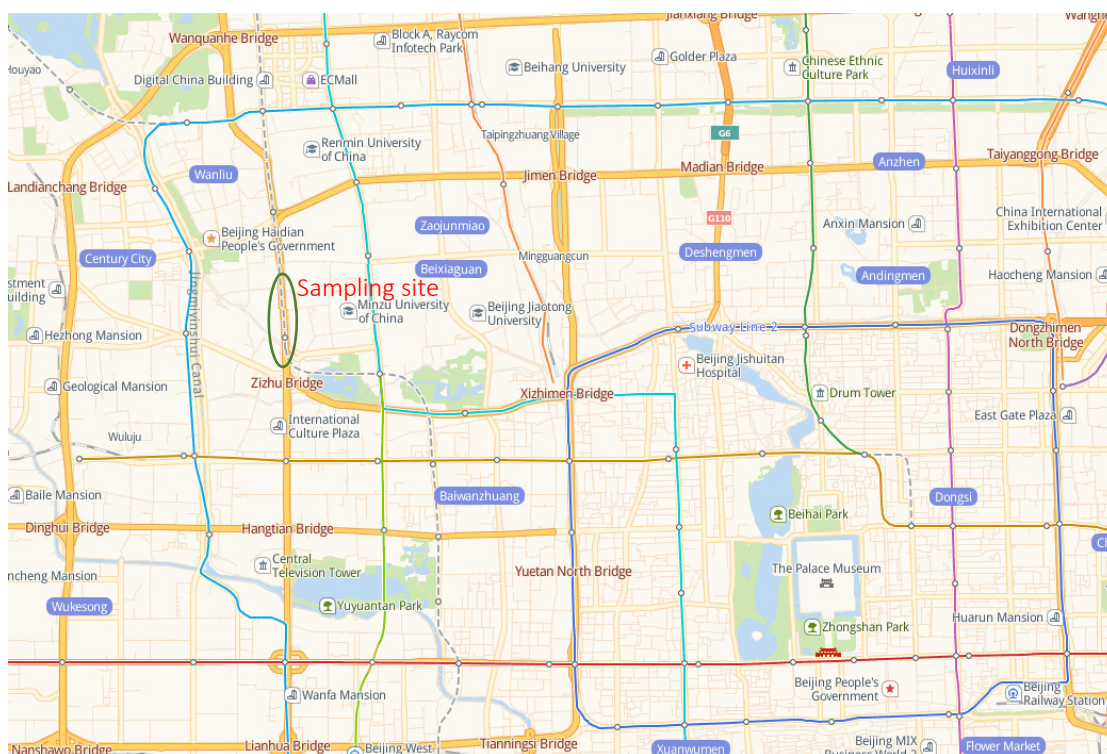

**Figure S1.** The location of tree leaves collected.

Supplement: Supplementary file 1 [file toxics-10-00634-s001.zip › toxics-1944535-supplementary.pdf]
